# PySmooth: a Python tool for the removal and correction of genotyping errors

**DOI:** 10.1186/s13104-024-06753-4

**Published:** 2024-04-11

**Authors:** Benjamin Soibam, Gregg Roman

**Affiliations:** 1 Department of computer science and engineering technology, University of Houston- Downtown, Houston, TX One Main St, 77002 USA; 2 Department of Biomolecular Sciences, School of Pharmacy, University of Mississippi, 415W Faser Hall, University, Oxford, Mississippi, MS 38677-1848 USA

**Keywords:** SNPS, QTLs, SMOOTH, genotype mapping and correction

## Abstract

**Summary:**

In genetic mapping studies involving many individuals, genome-wide markers such as single nucleotide polymorphisms (SNPs) can be detected using different methods. However, it comes with some errors. Some SNPs associated with diseases can be in regions encoding long noncoding RNAs (lncRNAs). Therefore, identifying the errors in genotype file and correcting them is crucial for accurate genetic mapping studies. We develop a Python tool called PySmooth, that offers an easy-to-use command line interface for the removal and correction of genotyping errors. PySmooth uses the approach of a previous tool called SMOOTH with some modifications. It inputs a genotype file, detects errors and corrects them. PySmooth provides additional features such as imputing missing data, better user-friendly usage, generates summary and visualization files, has flexible parameters, and handles more genotype codes.

**Availability and implementation:**

PySmooth is available at https://github.com/lncRNAAddict/PySmooth.

**Supplementary Information:**

The online version contains supplementary material available at 10.1186/s13104-024-06753-4.

## Introduction

SMOOTH is a statistical method for the successful removal of genotyping errors from high-density genetic linkage maps [1]. In high-density genetic maps, a genotyping error is exhibited as a singleton [1, 2], which is a locus with an assigned genotype different from its neighboring loci. SMOOTH uses a simple statistical method to identify singleton and this approach is still used in current studies [3–5] before performing Quantitative trait loci (QTL) analysis. However, SMOOTH has various drawbacks that prevent a more user-friendly experience. The code was written in PASCAL which is not very user-friendly. Only three genotype codes are allowed in the input genotype file for SMOOTH: ‘A’ for homozygous, ‘B’ for heterozygous, and ‘U’ for missing data. There are cases when a genotype file that contains four genotype codes (for example recombinant lines which are descendants of two different parents: parents 1 and 2) representing homozygous parent 1, homozygous parent 2, heterozygous, and missing data. This can’t be handled by SMOOTH directly because SMOOTH doesn’t differentiate between homozygous parent 1, homozygous parent 2. To run SMOOTH for such files, one can apply SMOOTH in two different ways. One approach is to assign homozygous parents 1 and 2 the same genotype code ‘A’. The second approach is to treat genotype code of one homozygous parent as missing labels and apply SMOOTH. This will be repeated by masking the other homozygous parent labels. These are not ideal ways to correct genotype error or identify singletons because the genotype codes representing the homozygosity of the other parental map is ignored. Original SMOOTH doesn’t generate visualizations and summary files that reports the number and locations of detected singletons. SMOOTH assigns a score that represents the probability that the marker is a singleton based on the genotype calls of the marker’s neighbors. An initial threshold is applied to the score to identify singletons. It goes through an iterative process where in each step a new score is assigned, and the threshold is decreased at each iteration until a lower threshold is reached [1]. SMOOTH implementation doesn’t allow the user to test different threshold values. The user must manually separate genotype file into multiple files, each file representing a unique chromosome.

Here, we present a Python implementation of SMOOTH called PySmooth which offers an easy-to-use command line interface and solves the drawbacks mentioned above. PySmooth reads the input genotype file and identifies singletons based on the algorithm described in SMOOTH with some modifications to allow four genotype codes, and flexible parameters. Unlike SMOOTH which doesn’t correct the singletons and missing data, PySmooth corrects genotype errors using a k-nearest algorithm [6]. At each step, PySmooth generates summary files and visualizations that can be inspected by the user for further interpretation.

## Main text

### Materials and methods

PySmooth was implemented in Python 3.8. The command-line interface software takes a genotype file as input identifies singletons, and imputes missing data and singletons based on the k-nearest neighbor algorithm [6]. Unlike SMOOTH, the user doesn’t have to create separate genotype files for each unique chromosome. PySmooth detects the unique number of chromosomes and runs the algorithm separately on each chromosome. The user also has the option to provide a list of chromosomes to be processed. PySmooth primarily processes the input file in three stages.

**Fig. 1:**
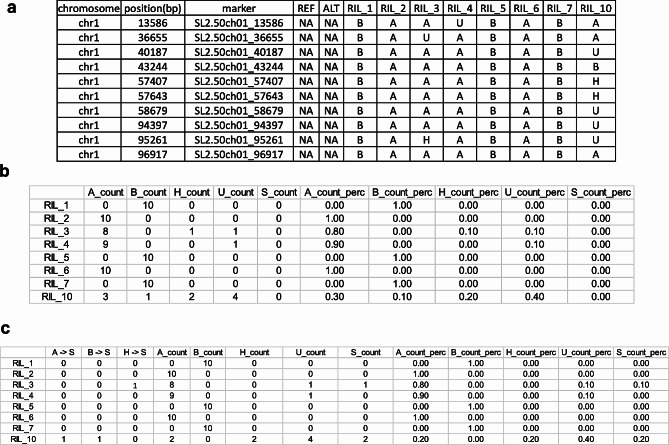
Example files of PySmooth. (**a**) Example input genotype file. The first row is the header. Each subsequent row represents a unique marker. The first three columns represent the chromosome name, location, and identification name of the marker, respectively. The fourth and fifth column represent reference/major allele and alternate allele, respectively. These columns can be left blank if not known. A heatmap representing this genotype file is shown in Fig. 2a. (**b**) Example statistics file corresponding the input genotype file. For each sample the frequency and percentage of each type of genotype call are reported (A: homozygous 1, B: homozygous 2, H: heterozygous, U: missing). A corresponding bar plot is shown in Fig. 2d. (**c**) Example file indicating the number of singletons detected. In each sample (row), the number and percentage of singletons detected (‘S_count’ and ‘S_count_perc’) are reported. Also reported are the number of original A, B, H genotype calls which were detected as Singletons (S)

**Fig. 2:**
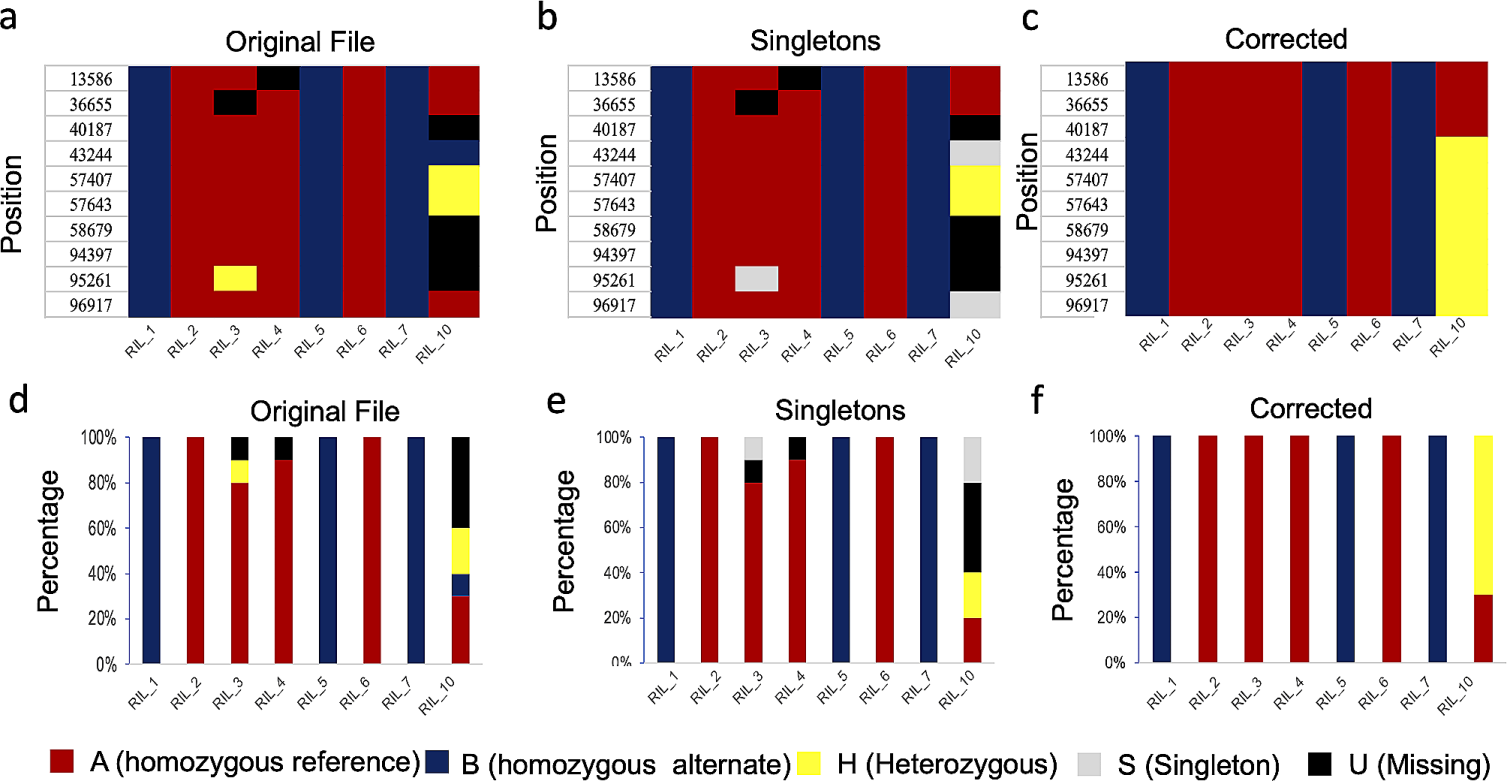
Example color-coded heatmap and bar plots generated by PySmooth. (**a**) Color-coded heatmaps of the original genotype file from Fig. 1a. After singletons identification by PySmooth, the heatmap is shown in (**b**), and after genotype correction, the heatmap is shown in (**c**). The heatmap in (**c**) doesn’t contain singletons and missing labels. Each column in the heatmap represents a sample, the rows represent the markers. Corresponding bar plots of original genotype file, after singletons identification, and correction are shown in (**d**), (**e**), and (**f**), respectively. The y-axis represents the percentage of each genotype label in the samples. A, B, H, S, and U represent reference parent homozygous, alternate parent homozygous, heterozygous, singletons, and missing data, respectively

In the first stage, PySmooth inspects the input genotype file (Fig. 1A), generates a summary text file (Fig. 1B) and a bar plot that summarize the statistics of different genotype codes in the genotype file. It also generates a heatmap plot of the samples, where genotype codes are uniquely color-coded (Fig. 1). In the second stage, PySmooth assigns a singleton score (*S*_*i*_) to a marker locus *i* by comparing the genotype code at locus *i* with genotype codes within a defined number of loci L flanking locus *i* on either side. 30 loci closest to locus *i* were used (15 loci to the left and 15 to the right). Therefore, L can be written as L = {*j: j < = 15*, and *j ≠ i*}. Singleton score (*S*_*i*_) to a marker locus *i* is defined as:

$$ \frac{\sum _{j L}{{y}_{ij}w}_{j }}{\sum _{j L}{w}_{j}}$$

with, *y*_*ij*_= 1 if the markers at locus *i* and locus *j* have different genotype codes assigned and *w*_*j*_ are the weights assigned to the flanking neighbors. SMOOTH uses a similar formula to compute the singleton score but its implementation only allows three genetic codes in the input file [1]. Same values of the weights used in SMOOTH [1] are used in PySmooth. Like SMOOTH, a high threshold of 0.99 is used to identify singletons from the singleton scores. It goes through a sequence of iterations with decreasing thresholds by gap of 0.02 until 0.70 is reached. Unlike SMOOTH, PySmooth provides the user an option to input the thresholds and the gap allowing experimentation with different values for thresholds and gap. After the second stage where the singletons are identified, PySmooth generates a new genotype file with the singletons marked as “S” and summary file that indicates how many of each original genotype code were switched to singleton “S” (Fig. 1C). A bar plot, a summary file and a heatmap (Fig. 2) are also generated after the second stage. In the third stage, PySmooth imputes the missing genotype and the singleton using a k-nearest neighbor algorithm [6] with a default value of k = 30. The user has the option to adjust this parameter. The default value of 30 is chosen because 30 closest neighbors are used to score singletons in PySmooth. After the third stage, PySmooth generates the corrected genotype file along with bar plot, heatmap, and a summary file. The summary files generated by PySmooth can be opened as excel spreadsheets and investigated further by the users.

### Usage and examples

PySmooth can run on windows, Linux, MacOs, and computing cluster systems with python and required python dependencies installed. PySmooth offers an easy-to-use command line interface to run a complete analysis through one main script called “*run_smooth.py*”. The only required input to execute *run_smooth.py* is an input genotype file (e.g. my_genotype.csv). All output files will have a prefix “test” by default. To run PySmooth analysis, the user can simply execute the following python command.

python run_smooth.py -i my_genotype_file.csv

PySmooth also offers several options to control the name of the output files, the chromosomes to be analyzed, number of k-nearest neighbors, thresholds, and gap values for singletons identification as shown below with options -o, -c, -l, -u, -g, -k, respectively. An example command is shown below which will generate all output files with the prefix “my_output”.

python run_smooth.py -i my_genotype_file.csv -o my_output -c chr1,chr2,chr3 -l 0.80 -u 0.98 -g 0.02 -k 34

The above command executes PySmooth for three chromosomes chr1, chr2, and chr3, and uses the number of k-nearest neighbors, upper threshold, lower threshold, and gap values for singletons identification as 34, 0.98, 0.80, and 0.02 respectively.

### Example input file and outputs

We tested PySmooth on an example input genotype file named “my_genotype_file.csv” (Additional file 1). The first, second, and third columns indicate the chromosome name, position, and name of the marker, respectively. The fourth and the fifth column indicates the “reference” allele and the alternate allele, respectively. If there is no information for these two columns, they can be left blank or filled with “NA”. The subsequent columns indicate the genotype calls of the samples. Four genotype codes can be used. A, B, H, and U represent reference parent homozygous, alternate parent homozygous, heterozygous, and missing data, respectively.

If the command “python run_smooth.py -i my_genotype_file.csv -o my_output” is executed, since this specific input genotype file contains only one chromosome (chr1), PySmooth generates three output summary files (Additional file 1) that contain percentages of homozygous, heterozygous calls for each individual for the raw genotype file, after singleton detection, and after error correction, respectively. The files not only indicate the number of singletons detected in each sample but also the fraction from each category of genotype calls detected as singletons. Three bar plot files (Additional file 1), that visualize the output summary files are also generated. Three heatmap files (Additional file 1) are also generated that visualize a color-coded image of different genotype codes in the original file, after singleton detection, and after error correction, respectively. Finally, PySmooth also outputs the genotype file with singletons and the final genotype file after correction of the singletons.

### PySmooth versus SMOOTH

To compare the accuracy of PySmooth and SMOOTH in predicting singletons in a genotype file, we simulated an F_2_ population consisting of 120 individuals and six chromosomes with 5720 marker locations. Using a Poisson distribution of λ = 1, recombination breakpoints were introduced in the F_2_ population. Each marker locus was labeled with one the three possible genotypes: ‘A’ (homozygous where the allele is inherited from one parent), ‘H’ (heterozygous locus with alleles from both parents), and ‘B’ (alternative homozygous locus where the allele is inherited from the other parent). Errors and missing values were introduced to the genotype file that represent the F_2_ population by randomly mislabelling 20% of the loci and marking another 10% of the loci as missing. PySmooth and SMOOTH were both applied to the same genotype file that contains errors and missing values. Two metrics were computed: percentage of introduced errors which were correctly predicted as singletons and percentage of correctly labeled loci which were incorrectly predicted as singletons. An accurate tool should have a higher and lower value of the first and the second metric, respectively. We found that PySmooth achieved superior accuracy by correctly predicting 96.64% of the introduced errors as singletons compared to 73.32% by SMOOTH. PySmooth predicted only 0.03% of the correct genotype labels as singletons compared to 24% misprediction by SMOOTH. When the error rate was increased to 30%, PySmooth was able to recover 82.47% of the introduced errors as singletons compared to 68% by SMOOTH. These results show that PySmooth performs better than SMOOTH.

### Limitations

Our tool PySmooth offers several improvements over the SMOOTH tool by allowing a user-friendly command interface, summary and visualization files, more genotype codes, flexible parameters, and correcting genotype errors.

The main limitation of the current version of PySmooth is the lack of the feature of parallel processing to reduce computation time in large genotype files. One can leverage multiple cores and process different chromosomes simultaneously to reduce computation time. However, this can be overcome manually with the current version of PySmooth. Users can execute PySmooth in computing clusters or systems with multiple cores for different chromosomes simultaneously in parallel to reduce computing time. For this, the user must manually create different files that correspond to different chromosomes and execute PySmooth on these files separately. In the future version of PySmooth, an automated multi-core processing feature will be incorporated.

There is only feature of the marker in a genotype file which is its position. In the genotype file, markers which are close to each other will most likely have the same genotype call. Therefore, K-nearest neighbor was used to impute missing data because of its reliance on distance between data points. In future versions of PySmooth, we plan to add more methods which the user can choose from.

### Electronic Supplementary Material

Below is the link to the electronic supplementary material.

Additional file 1

## Data Availability

PySmooth is available at https://github.com/lncRNAAddict/PySmooth.
