# Supplementary figures and images for: PySmooth: a Python tool for the removal and correction of genotyping errors
Source: BMC Res Notes. 2024 Apr 11;17:103. doi: 10.1186/s13104-024-06753-4 (PMC11010338; doi:10.1186/s13104-024-06753-4)

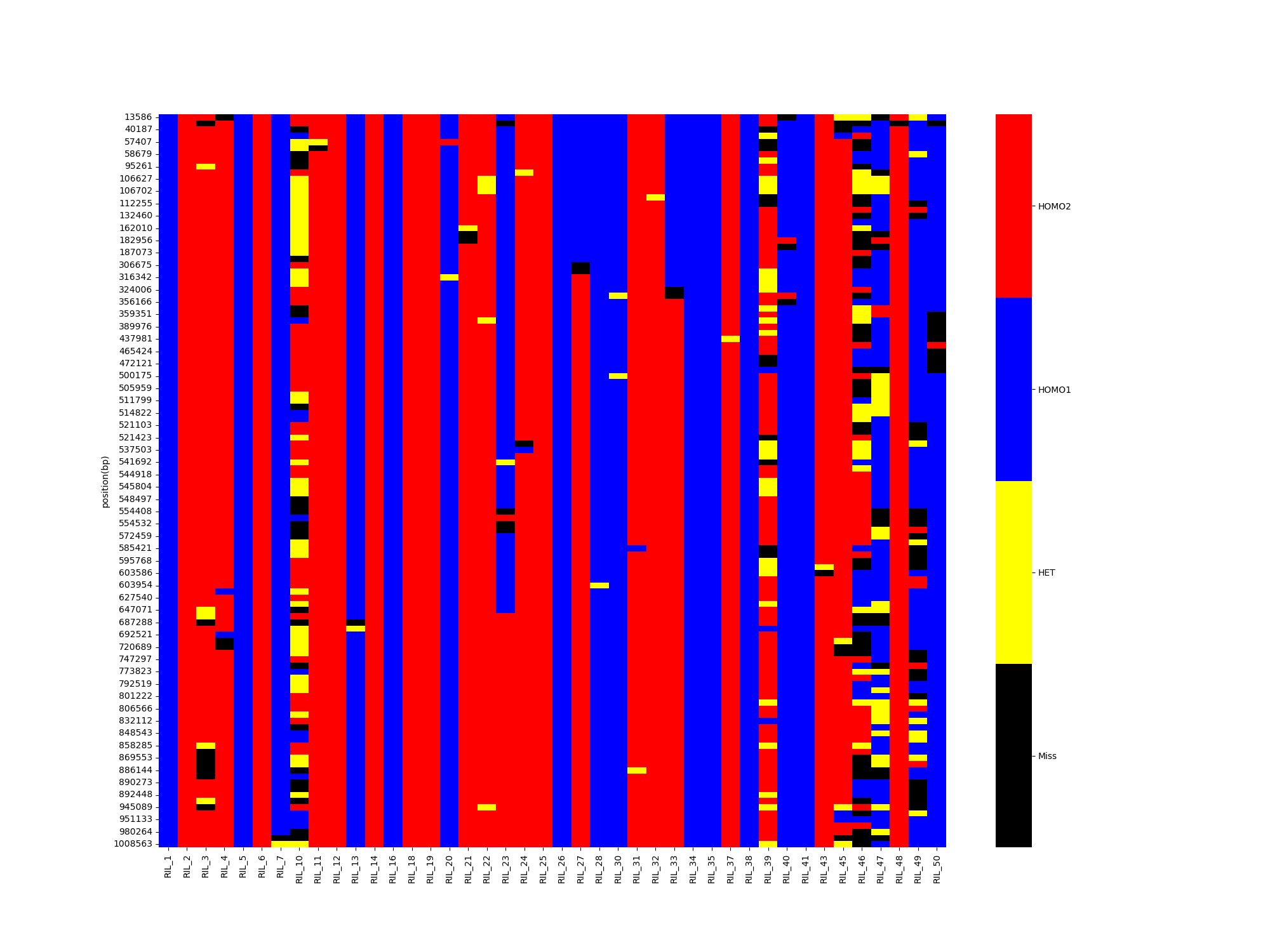

Supplement: Supplementary file 1 — Additional file 1 [file 13104_2024_6753_MOESM1_ESM.zip › Example_input_output/my_output_chr1.heatmap.png]

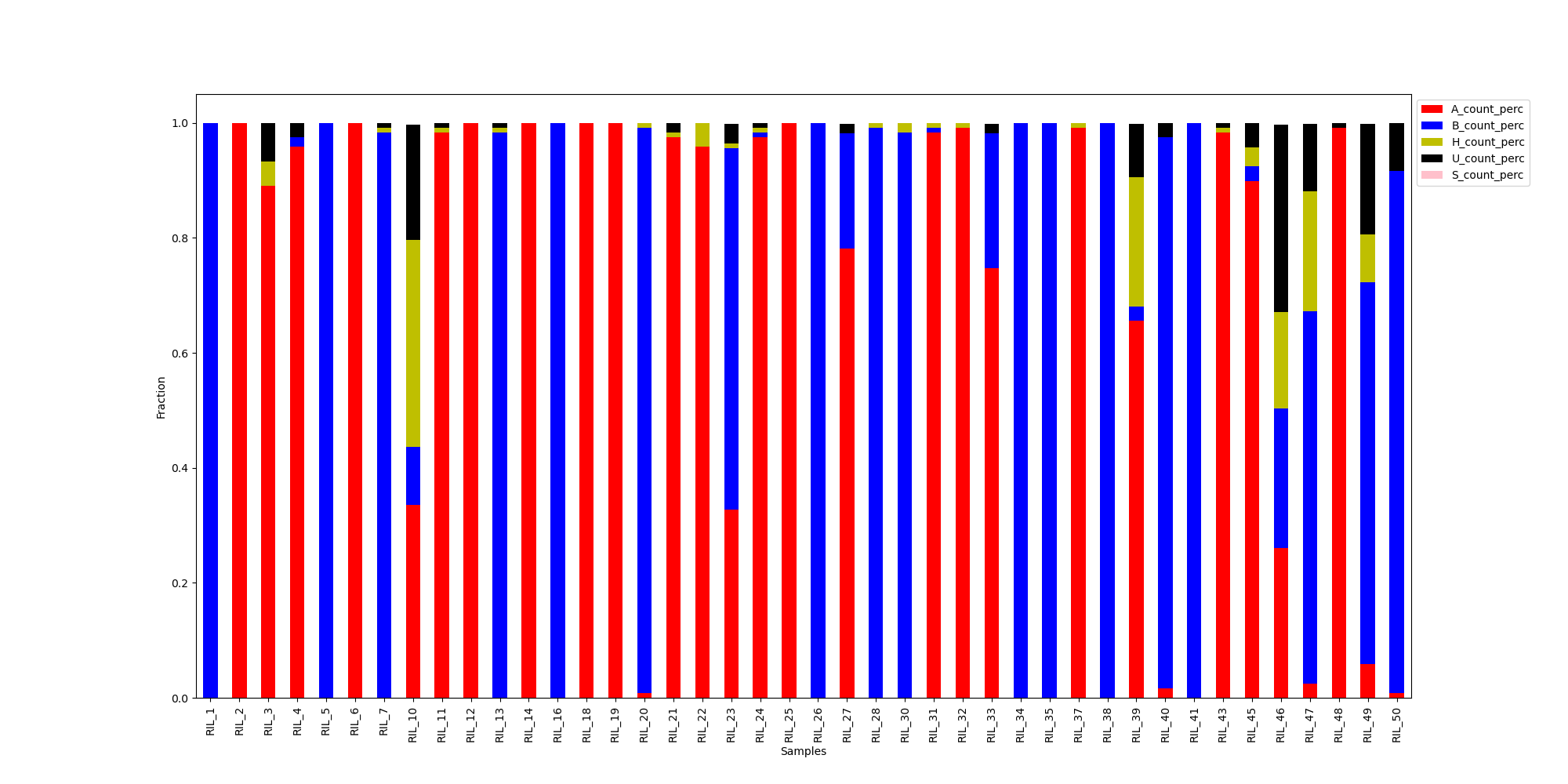

Supplement: Supplementary file 1 — Additional file 1 [file 13104_2024_6753_MOESM1_ESM.zip › Example_input_output/my_output_chr1.stats.png]

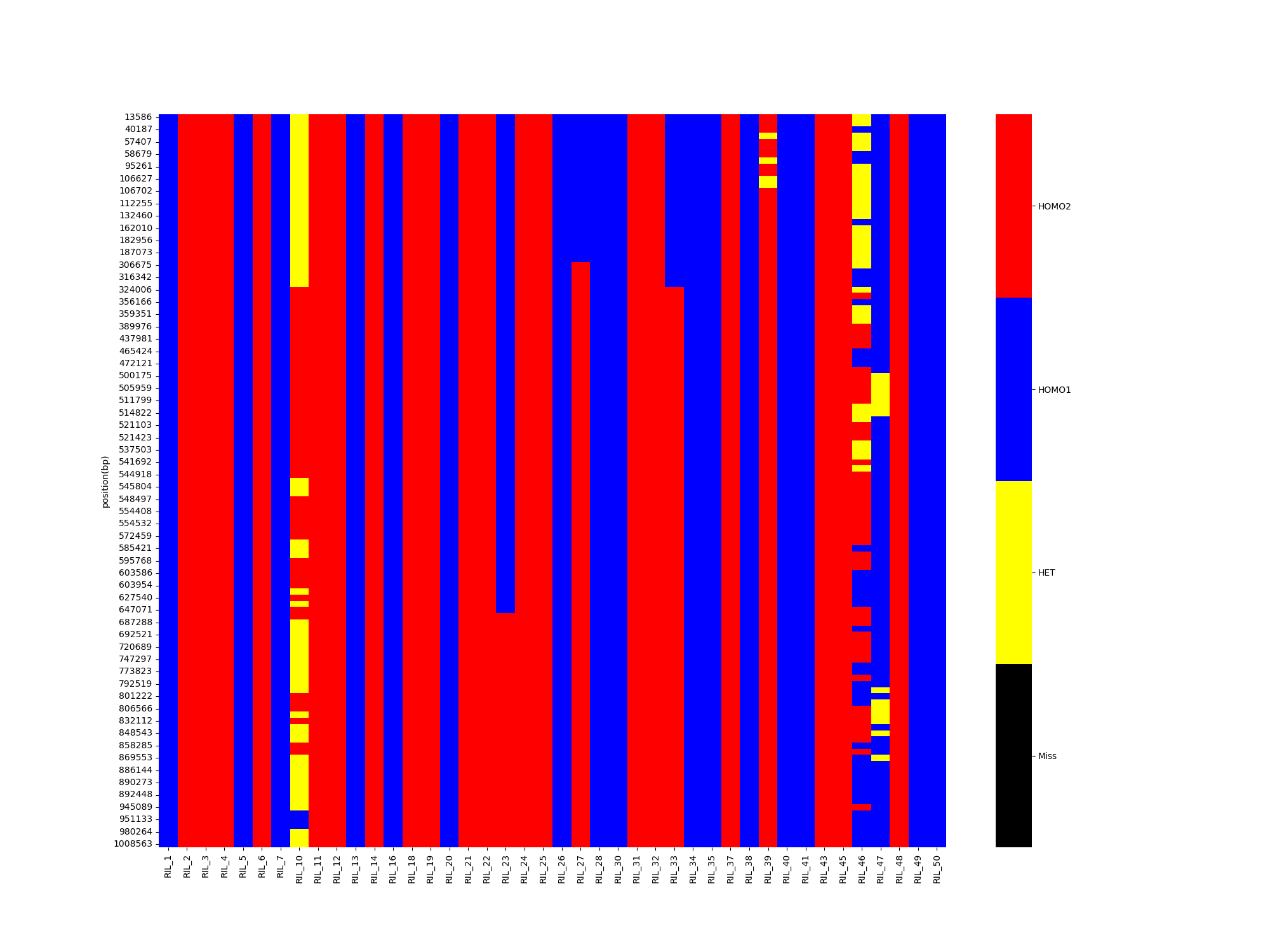

Supplement: Supplementary file 1 — Additional file 1 [file 13104_2024_6753_MOESM1_ESM.zip › Example_input_output/my_output_chr1_imputed_heatmap.png]

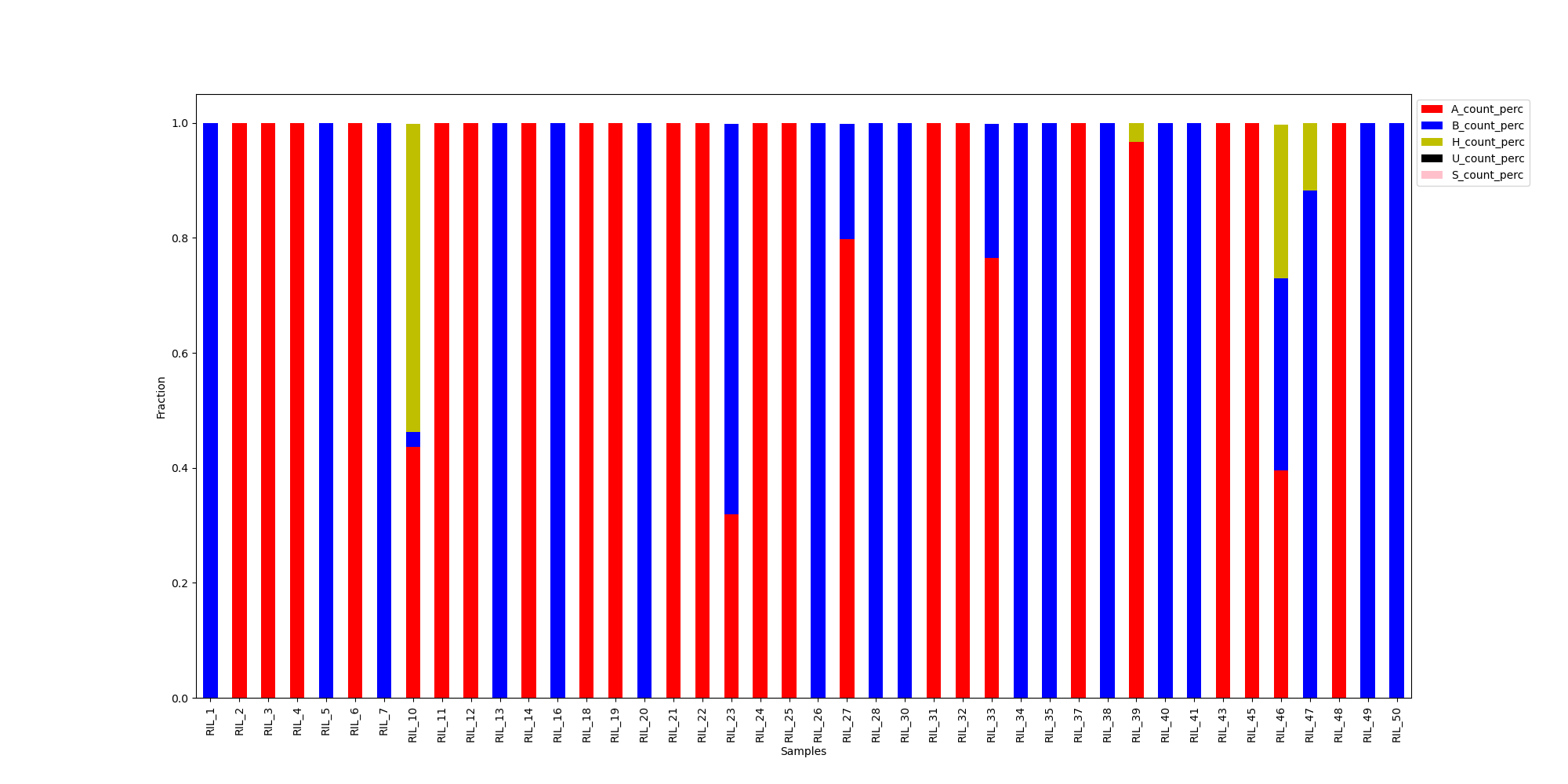

Supplement: Supplementary file 1 — Additional file 1 [file 13104_2024_6753_MOESM1_ESM.zip › Example_input_output/my_output_chr1_imputed_stats.png]

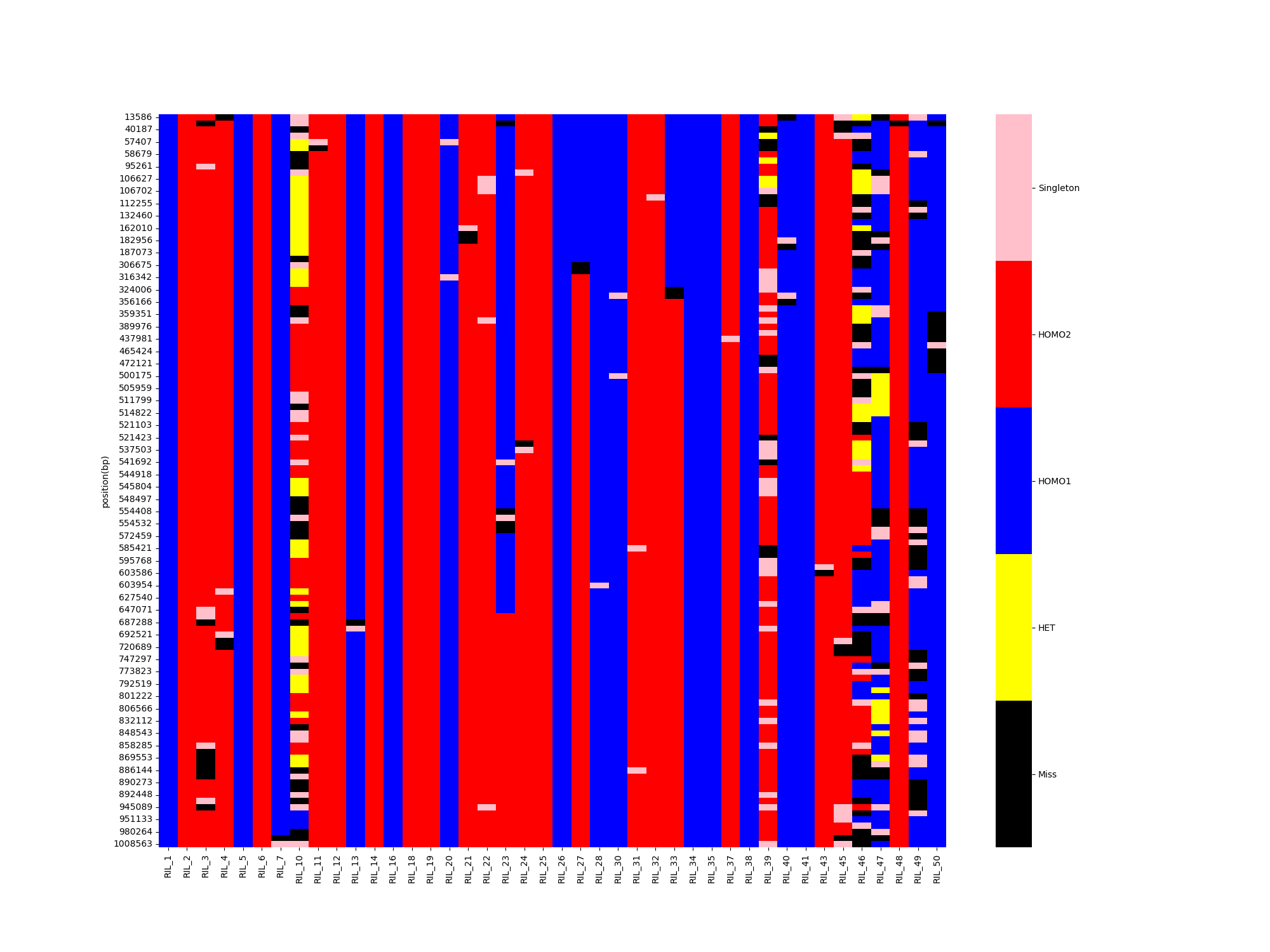

Supplement: Supplementary file 1 — Additional file 1 [file 13104_2024_6753_MOESM1_ESM.zip › Example_input_output/my_output_chr1_singletons_heatmap.png]

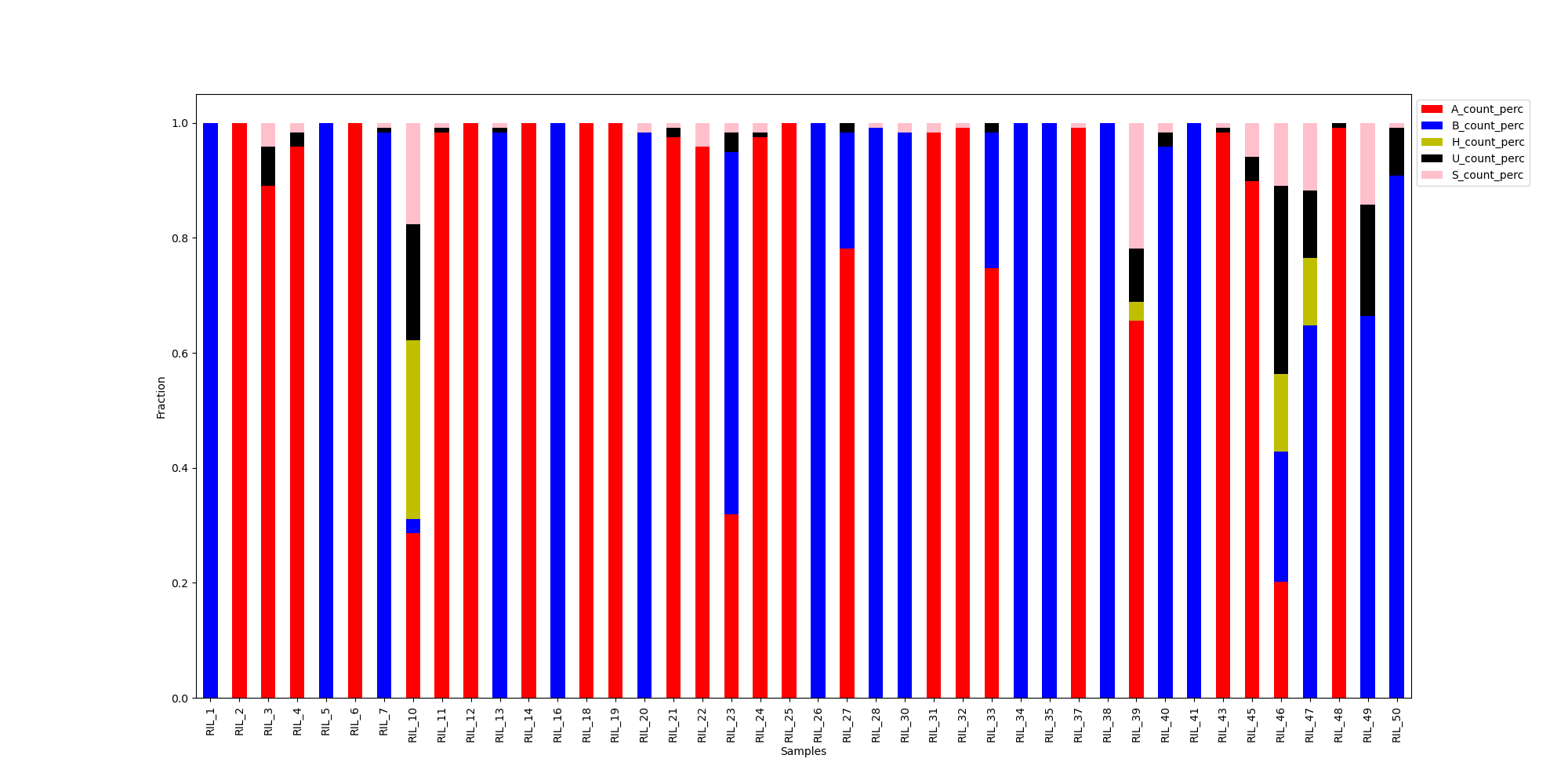

Supplement: Supplementary file 1 — Additional file 1 [file 13104_2024_6753_MOESM1_ESM.zip › Example_input_output/my_output_chr1_singletons_stats.png]
